# Sex in the PAC: A hidden affair in dark septate endophytes?

**DOI:** 10.1186/1471-2148-11-282

**Published:** 2011-09-30

**Authors:** Pascal L Zaffarano, Valentin Queloz, Angelo Duò, Christoph R Grünig

**Affiliations:** 1 Institute of Integrative Biology (IBZ), Forest Pathology and Dendrology, ETH Zurich, 8092 Zürich, Switzerland; 2 Microsynth AG, Schützenstrasse 15, 9436 Balgach, Switzerland

## Abstract

**Background:**

Fungi are asexually and sexually reproducing organisms that can combine the evolutionary advantages of the two reproductive modes. However, for many fungi the sexual cycle has never been observed in the field or *in vitro *and it remains unclear whether sexual reproduction is absent or cryptic. Nevertheless, there are indirect approaches to assess the occurrence of sex in a species, such as population studies, expression analysis of genes involved in mating processes and analysis of their selective constraints. The members of the *Phialocephala fortinii *s. l. - *Acephala applanata *species complex (PAC) are ascomycetes and the predominant dark septate endophytes that colonize woody plant roots. Despite their abundance in many ecosystems of the northern hemisphere, no sexual state has been identified to date and little is known about their reproductive biology, and how it shaped their evolutionary history and contributes to their ecological role in forest ecosystems. We therefore aimed at assessing the importance of sexual reproduction by indirect approaches that included molecular analyses of the mating type (*MAT*) genes involved in reproductive processes.

**Results:**

The study included 19 PAC species and > 3, 000 strains that represented populations from different hosts, continents and ecosystems. Whereas *A. applanata *had a homothallic (self-fertile) *MAT *locus structure, all other species were structurally heterothallic (self-sterile). Compatible mating types were observed to co-occur more frequently than expected by chance. Moreover, in > 80% of the populations a 1:1 mating type ratio and gametic equilibrium were found. *MAT *genes were shown to evolve under strong purifying selection.

**Conclusions:**

The signature of sex was found in worldwide populations of PAC species and functionality of *MAT *genes is likely preserved by purifying selection. We hypothesize that cryptic sex regularely occurs in the PAC and that further field studies and *in vitro *crosses will lead to the discovery of the sexual state. Although structurally heterothallic species prevail, it cannot be excluded that homothallism represents the ancestral breeding system in the PAC.

## Background

The origin and maintenance of sexual reproduction is a controversially discussed topic in evolutionary biology as reflected by the multitude of theories that have been proposed to explain why sexual reproduction, although highly costly, is widely occurring in nature [1-6]. Asexual organisms have a two-fold advantage over sexual conspecifics and can effectively disseminate [7-10]. In contrast, sexual reproduction efficiently eliminates deleterious mutations [11] and creates genetic variation that favors natural selection and accelerates adaptation to changing environments [1]. However, many species are capable of reproducing both sexually and asexually and illustrate how difficult it is to provide a general explanation on the evolutionary significance of sex. Fungi combine the advantages of the two reproductive modes. Several reasons were proposed for why the cost of sex compared to asex is lower in fungi than for animals and plants [12] because (i) fungi can be isogamous and thus the contribution of resources to the zygote by the gametes is limited and (ii) many fungi are also homothallic (self-fertile) and do not depend on finding a compatible mate which reduces the cost of sex, whereas others are heterothallic (self-sterile) and mating is regulated by mating type factors. (iii) Moreover, the majority of fungi can alternate between asexual and sexual reproduction and thus sexuality can be adjusted to when opportunity costs are low, for example at the end of the growing season of a host plant on which a fungus is dependent when adverse conditions are disadvantageous for somatic growth. Sex is also linked to essential processes such as the formation of resistant spores that are able to survive unfavourable conditions and enable new genotypes to be spread into new environments. Once the link between sex and such essential processes has evolved, selection against frequent sexual recombination might be less effective.

However, for many fungi, especially for filamentous ascomycetes, only part of their life cycle is known. These taxa are classified as Deuteromycota or "fungi imperfecti" due to the lack of sexual morphology [13], but it is unclear whether sexual reproduction is absent, rare or cryptic because sexual morphology is often difficult to observe in nature or in the laboratory [14,15]. Thus, the importance of sexual reproduction in natural populations of such species remains an open question which can be addressed by direct and indirect approaches [16].

The direct apporach consists in searching for the sexual state (teleomorph) in the field or in the laboratory. However, it is often difficult to induce the teleomorph *in vitro *as many factors (e.g. nutrient media, temperature, light exposure, selection of compatible mating types) need to be optimized for a successful induction [17,18]. Moreover, although sexual structures obtained in the laboratory indicate that the ability for sexual reproduction has not been lost, its importance in the field remains to be established by population studies and by monitoring the teleomorph in the field [16]. Indirect approaches comprise population studies that test the null hypothesis of random mating and include analysis of gametic disequilibrium, mating type ratios, genotypic diversity and phylogenetic analysis [19,20]. Moreover, functionality of genes involved in mating processes can be tested by expression analyses and provide further evidence for sexual reproduction [21]. Alternatively, estimates of selective pressures acting on such genes might indicate whether functionality is preserved [22-26].

In this study we aimed at elucidating the importance of sexual reproduction in the *Phialocephala fortinii *sensu lato - *Acephala applanata *species complex (PAC) that belongs to the dark septate endophytes, a polyphyletic group of ascomycetes with characteristic melanized, septate hyphae that commonly colonize roots of woody plant species [27]. The PAC is composed of more than 20 species, eigth of which were formaly described [28,29], and they dominate the endophytic assemblages in roots of conifers and members of the *Ericaceae *in the northern hemisphere from polar to subtropical regions [30-32]. PAC species form communities of up to ten species [32,33] and species abundance distributions within these communities ususally follow a hyperbolic distribution with a few abundant species and many "rare" species, consistent with the community structures of many other organismal groups [34]. Interestingly, no biogeographical pattern was found for PAC species and community compositions did neither correlate with composition of the host species nor with climate [32] supporting the the hypothesis that "everything is everywhere" [35]. Despite the broad geographical occurrence of this fungal species complex, the teleomorph has never been observed for any of its species.

In ascomycetes, the mating type *(MAT) *locus exercises key regulatory functions involved in mating processes and usually defines homothallism and heterothallism [36,37]. In heterothallic ascomycetes the *MAT *locus is characterized by two alternative forms, *MAT1-1 *and *MAT1-2*, called idiomorphs because they contain dissimilar DNA sequences at the same chromosomal location. These encode proteins with conserved DNA binding domains that are involved in transcription regulation leading to the attraction of compatible mating types in order to initiate the mating process [37] and are also involved in controlling the regulation of internuclear recognition in later steps of sexual development [38-40]. In contrast, in homothallic species, a single individual generally contains all of the *MAT *genes and is thus capable of selfing [41]. In filamentous ascomycetes the simpliest idiomorphs consist of *MAT1-1 *that carries one gene called *MAT1-1-1 *encoding a protein with an α-domain binding motif and *MAT1-2 *that contains one gene called *MAT1-2-1 *encoding a protein with a high mobility group (HMG)-binding domain [42,43]. However, additional *MAT *genes are regularely found in ascomycetous species [42]. For example, in the helotialean species *Pyrenopeziza brassicae *and *Rhynchosporium secalis *that are closely related to the PAC [44] up to two additional *MAT genes *have been identified in the *MAT1-1 *idiomorph. One is designated *MAT1-1-3*, encodes a protein with an HMG-binding domain and is present in both species, whereas *MAT1-1-4 *encodes a metallothionein-like protein and is only present in *P. brassicae *[45,46].

In a recent study we cloned the *MAT *genes of eight PAC species [44]. and expected the *MAT *idiomorphs to be similar to those of their helotialean relatives. Seven examined species showed a heterothallic organization of the *MAT *locus because strains either contained the *MAT1-1 *idiomorph carrying *MAT1-1-1 *and *MAT1-1-3 *or the *MAT1-2 *idiomorph carrying *MAT1-2-1*. In contrast, in *A. applanata *the *MAT *locus structure was indicative of homothallism because all of the three *MAT *genes were present in single strains. However, it was neither possible to induce sexual reproductive structures in crossing experiments nor to show that *MAT *genes were expressed under laboratory conditions [44]. In contrast, analysis of multi-locus gametic disequilibrium for a limited number of Swiss populations of four PAC species showed that in most populations the index of association I_A _[47] did not deviate significantly from zero which is indicative of recombination [33,48]. Because PAC species are successful root colonizers of a multitude of plant species in many ecosystems, we hypotesize that sexual reproduction occurrs as it confers the ability to successfully adapt to different environments worldwide.

In the present study we aimed at gaining a more complete picture about the importance of sexual reproduction in this species complex. In particular, we determined (i) the *MAT *locus structure for 11 additional PAC species, (ii) whether opposite mating types in populations of the PAC species deviated from the 1:1 ratio expected under random mating, (iii) the deviation from gametic equilibrium in the fungal populations, (iv) the spatial distribution of mating types at the collection sites, and (v) the selective pressures acting on *MAT *genes.

## Results

### Organization of the *MAT *locus in PAC species

**Figure 1:**
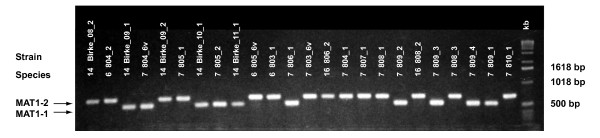
**Example of multiplex PCR**. Multiplex PCR amplification of idiomorph specific bands in selected strains of different PAC species, using the primers Pf_HMG_R.03, Pf_HMG_F4 and Pf_MAT1-1F1c.

The multiplex PCR amplified either a *MAT1-1 *specific fragment of ~550 bp or a *MAT1-2 *specific fragment of ~750 bp in strains of any PAC species, except in the homothallic *A. applanata *for which no fragments were amplified, as expected due to its *MAT *locus organization [44] (Figure 1). During the screening of the collections, occasionally (in < 2.5% of the screened strains) both fragments were amplified in single strains, indicating potential homothallism, and in < 1.0% of the strains an amplification failure of the PCR product was recorded. However, re-analyzing newly prepared single-hyphal-tip cultures from a subset of eight of these strains, an amplification of single idiomorph-specific fragments was always obtained (see Additional file 1).

The *MAT *locus of 11 additional PAC species was sequenced and characterized for one *MAT1-1 *and *MAT1-2 *strain per species (Table 1), and its structure was found to be congruent with the *MAT *locus structure of the seven previously studied species [44]. The *MAT1-1 *idiomorphs contained the *MAT1-1-1 *and *MAT1-1-3 *gene whereas the *MAT1-2 *idiomorphs included the *MAT1-2-1 *gene.

**Table 1:** Strains for which the complete mating type idiomorph was sequenced

Species	Strain	Idiomorph	Geographic origin	Host/Substrate^1^	Reference/Collector	**GenBank accession No**.
*Phialocephala turicensis*	1_120_2	*MAT1-1*	Zürichberg, Switzerland	root of *Picea abies*	Zaffarano et al. (2010)	[GenBank:HM347273]
	1_117_3	*MAT1-2*	Zürichberg, Switzerland	root of *Picea abies*	Zaffarano et al. (2010)	[GenBank:HM347292]
*Phialocephala letzii*	2_120_3	*MAT1-1*	Zürichberg, Switzerland	root of *Picea abies*	Zaffarano et al. (2010)	[GenBank:HM347274]
	2_116_2	*MAT1-2*	Zürichberg, Switzerland	root of *Picea abies*	Zaffarano et al. (2010)	[GenBank:HM347293]
*Phialocephala europaea*	3_136_1	*MAT1-1*	Zürichberg, Switzerland	root of *Picea abies*	Zaffarano et al. (2010)	[GenBank:HM347275]
	3_122_3	*MAT1-2*	Zürichberg, Switzerland	root of *Picea abies*	Zaffarano et al. (2010)	[GenBank:HM347294]
*Phialocephala helvetica*	4_140_4	*MAT1-1*	Zürichberg, Switzerland	root of *Picea abies*	Zaffarano et al. (2010)	[GenBank:HM347276]
	4_153_2	*MAT1-2*	Zürichberg, Switzerland	root of *Picea abies*	Zaffarano et al. (2010)	[GenBank:HM347295]
*Phialocephala uotolensis*	5_265_3	*MAT1-1*	Uetliberg; Switzerland	root of *Picea abies*	Zaffarano et al. (2010)	[GenBank:HM347277]
	5_265_4	*MAT1-2*	Uetliberg; Switzerland	root of *Picea abies*	Zaffarano et al. (2010)	[GenBank:HM347296]
*Phialocephala subalpina*	6_30_4	*MAT1-1*	Bödmeren, Switzerland	root of *Picea abies*	Zaffarano et al. (2010)	[GenBank:HM347278]
	6_37_6v	*MAT1-2*	Bödmeren, Switzerland	root of *Vaccinium myrtillus*	Zaffarano et al. (2010)	[GenBank:HM347297]
*Phialocephala fortinii *s.s.	7_K93_444^a^	*MAT1-1*	Suonenjoki, Finland	roots of *Pinus sylvestris*	Zaffarano et al. (2010)	[GenBank:HM347279]
	7_6_7v	*MAT1-2*	Bödmeren, Switzerland	root of *Vaccinium myrtillus *	Zaffarano et al. (2010)	[GenBank:HM347298]
CSP8	8_L_05-7	*MAT1-1*	Sheep Creek, USA	n.a.	J.B. Zaerr	[GenBank:HM347280]
	8_L_02-5	*MAT1-2*	Sheep Creek, USA	n.a.	J.B. Zaerr	[GenBank:HM347299]
CSP9	9_ST_43-1	*MAT1-1*	Walder Steinenberg, Switzerland	root of *Picea abies*	N. Brenn	[GenBank:HM347281]
	9_ST_02-3	*MAT1-2*	Walder Steinenberg Switzerland	root of *Picea abies*	N. Brenn	[GenBank:HM347300]
CSP10	10_CS_15L_2	*MAT1-1*	Court, Switzerland	root of Picea abies	C.R. Grünig	[GenBank:HM347282]
	10_CS_9_1	*MAT1-2*	Court, Switzerland	root of *Picea abies*	C.R. Grünig	[GenBank:HM347301]
CSP11	11_AF196-16	*MAT1-1*	Varena plantation, Lithuania	root of *Picea abies*	A. Menkis	[GenBank:HM347283]
	11_AF200-1	*MAT1-2*	Varena plantation, Lithuania	root of *Picea abies*	A. Menkis	[GenBank:HM347302]
CSP12	12_405_1	*MAT1-1*	Creux du Van, Switzerland	root of *Picea abies*	V. Queloz	[GenBank:HM347284]
	12_418_1	*MAT1-2*	Creux du Van, Switzerland	root of *Picea abies*	V. Queloz	[GenBank:HM347303]
CSP13	13_422_6v	*MAT1-1*	Creux du Van, Switzerland	root of *Vaccinium vitis-ideae*	V. Queloz	[GenBank:HM347285]
	13_414_2	*MAT1-2*	Creux du Van, Switzerland	root of *Picea abies*	V. Queloz	[GenBank:HM347304]
CSP14	14_426_4	*MAT1-1*	Creux du Van, Switzerland	root of *Picea abies*	V. Queloz	[GenBank:HM347286]
	14_444_6v	*MAT1-2*	Creux du Van, Switzerland	root of *Vaccinium myrtillus*	V. Queloz	[GenBank:HM347305]
CSP15	15_A_02-5	*MAT1-1*	Noonday, USA	n.a.	J. Hill	[GenBank:HM347287]
	15_A_08-4	*MAT1-2*	Noonday, USA	n.a.	J. Hill	[GenBank:HM347306]
CSP16	16_428_6v	*MAT1-1*	Creux du Van, Switzerland	root of *Vaccinium vitis-ideae*	V. Queloz	[GenBank:HM347288]
	16_404_2	*MAT1-2*	Creux du Van, Switzerland	root of *Picea abies*	V. Queloz	[GenBank:HM347307]
CSP18	18_410_1	*MAT1-1*	Creux du Van, Switzerland	root of *Picea abies*	V. Queloz	[GenBank:HM347290]
	18_439_1	*MAT1-2*	Creux du Van, Switzerland	root of *Picea abies*	V. Queloz	[GenBank:HM347308]
CSP19	19_1559-1	*MAT1-1*	Parco del Pollino, Italy	root of *Abies alba*	V. Queloz	[GenBank:HM347291]
	19_1545-1	*MAT1-2*	Parco del Pollino, Italy	root of *Abies alba*	V. Queloz	[GenBank:HM347309]

^1 ^n.a. = information not available

### Mating type ratios and gametic disequilibrium in PAC populations

The vast majority of populations (> 80%) did not deviate significantly from the expected 1:1 ratio in mating types (Table 2). After applying Bonferroni correction to adjust for errors of multiple comparisons (type 1 error), no significant deviations remained. Moreover, even those populations with < 10 individuals contained both mating types. Only a single population was found that did not include both mating types. Similarly, only six out of 52 populations showed significant gametic disequilibrium and these were restricted to populations of *P. subalpina *(Table 2), Furthermore, in only two of these *P. subalpina *populations both unequal mating type frequencies and gametic disequilibria were recorded.

**Table 2:** Mating type ratios for populations of species belonging to the *Phialocephala fortinii *s.l. - *Acephala applanata *species complex (PAC)

Species	Study site	Country	Marker type^1^	Number of strains	Mating type ratios				Gametic disequilibrium	
					Number of MLH	*MAT1-1*	*MAT1-2*	Significance^2^	**Number of MLH**^3^	I_A_^4^
*P. turicensis*	Rothwald	Austria	M	24	7	4	3	n.a.		
	Etang de la Gruère	Switzerland	M	203	12	9	3	*	12	0.28
	Uetliberg	Switzerland	RFLP	145	17	9	8		17	0.07
	Zürichberg	Switzerland	RFLP	48	13	7	6		13	0.13
*P. letzii*	Parabock	Switzerland	M	58	11	6	5		11	0.69
	Rothwald	Austria	M	46	16	7	9		16	-0.01
	Zürichberg	Switzerland	RFLP	52	18	13	5	*	18	0.12
*P. europaea*	Redwater	Canada	M	19	7	1	6	n.a.		
	Alaska	USA	M	19	12	7	5		13	0.41
	Catherine Creek	USA	M	17	8	5	3	n.a.		
	Sierra Nevada	USA	M	10	9	7	2	n.a.		
	Bödmeren	Switzerland	M	28	19	7	12		17	-0.18
	Scatlé	Switzerland	M	36	31	12	19		25	0.02
	Rothwald	Austria	M	86	40	23	17		38	-0.12
	Alpes Maritimes	France	M	77	43	24	19		34	0.05
	Creux du Van	Switzerland	M	123	51	27	24		43	-0.08
	Derberence	Switzerland	M	179	64	28	36		48	-0.05
	Runcaglia	Switzerland	M	95	45	22	23		41	0.02
	Zürichberg	Switzerland	RFLP	130	46	26	20		46	0.04
*P. helvetica*	Yuba	USA	M	25	5	3	2	n.a.		
	Ala Archa	Kirgistan	M	18	7	7	0	n.a.		
	Baikalsee	Russia	M	29	11	4	7		12	0.16
	Mansfield	Canada	M	63	16	4	12	*	16	0.46
	Sierra Nevada	USA	M	47	30	17	13		30	0
	Zürichberg	Switzerland	RFLP	40	15	11	4	*	15	-0.03
	Runcaglia	Switzerland	M	98	36	20	16		36	0.02
*P. uotolensis*	Alpes Maritimes	France	M	17	6	4	2	n.a.		
*P. subalpina*	Creux du Van	Switzerland	M	13	8	6	2	n.a.		
	Etang de la Gruère	Switzerland	M	15	14	8	6		14	0.05
	Massif Central/La Fage	France	M	70	30	14	16		29	-0.08
	Sheep Creek	USA	M	12	7	2	5	n.a.		
	Marsh Island	USA	M	23	13	3	10	*	13	0.17
	Pyrénées	France	M	19	13	5	6		13	0.92
	Redwater	Canada	M	12	11	3	8		10	-0.06
	Baikalsee	Russia	M	16	13	10	3	*	12	0.27
	Bialowieza	Poland	M	60	40	10	30	**	40	2.12***
	Noonday	USA	M	22	15	8	7		14	0.11
	Grass Creek	USA	M	28	22	11	11		21	-0.22
	Rothwald	Austria	M	43	23	12	11		23	0.70*
	Derberence	Switzerland	M	37	26	11	15		26	1.66***
	Valcartier	Canada	M	35	30	17	13		28	0.48
	Bödmeren	Switzerland	M	90	54	23	31		44	0.58
	Scatlé	Switzerland	M	118	74	42	32		71	0.71**
	Alpes Maritimes	France	M	125	75	46	29	*	75	0.58*
	Campolino	Italia	M	138	79	43	36		86	0.41
	Kevo	Finland	M	138	87	44	43		72	0.52*
*P. fortinii *s.s.	Glentanar	Scottland	M	10	5	3	2			
	Bödmeren	Switzerland	M	10	8	4	4			
	Marsh Island	USA	M	17	10	5	5		10	-0.17
	Noonday	USA	M	11	7	2	5			
	Hill of Fare	Scottland	M	23	15	9	6		13	-0.07
	Valcartier	Canada	M	23	17	8	9		15	0.37
	Etang de la Gruère	Switzerland	M	24	18	10	8		23	0.29
	Bialowieza	Poland	M	92	51	35	16	*	51	0.05
	La Fage	France	M	102	37	16	21		38	0.23
	Le Pirou	France	M	13	11	4	7		11	0.22
	Tschornohora	Ukraine	M	76	41	28	13	*	41	0.25
	Kevo	Finland	M	100	54	24	30		54	0.28
CSP8	Global	Global	M	42	20	9	11			
CSP9	Global	Global	M	10	5	4	1	n.a.		
CSP10	Global	Global	M	30	5	4	1	n.a.		
CSP11	Fuji	Japan	M	20	11	7	4		11	0.59
	Kevo	Finland	M	12	7	4	3	n.a.		
CSP12	Creux du Van	Switzerland	M	15	7	5	2	n.a.		
	Chychkan	Kirgistan	M	9	7	6	1	n.a.		
CSP13	Creux du Van	Switzerland	M	43	23	9	12		23	0.66
	Alaska	USA	M	14	10	7	3		11	0.41
CSP14	Kevo	Finland	M	16	14	4	10		14	-0.18
	Creux du Van	Switzerland	M	21	8	6	2	n.a.		
	Zinal_Baiting	Switzerland	M	72	26	14	12		25	-0.19
CSP15	Noonday	USA	M	28	14	6	8		15	-0.7
CSP16	Creux du Van	Switzerland	M	9	3	2	1	n.a.		
CSP18	Global	Global	M	20	4	1	3	n.a.		
CSP19	Global	Global	M	30	5	4	1	n.a.		

^1 ^Molecular markers used for clone-correction were 11 single-copy Restriction Fragment Length Polymorphisms (RFLP) or 12 microsatellite loci (M).

^2 ^n.a. = not available; significance test was not performed due to small sample size; * = significant deviation from a 1:1 ratio.

^3 ^MLH = Multilocus haplotypes inferred by RFLP or M; MLHs with missing data were excluded to calculate the index of association (I_A_).

^4 ^* = p < 0.05; ** = p < 0.01; *** = p < 0.001

### Spatial distribution of mating types in selected study sites

The spatial distribution of mating types was analyzed for four PAC species in ten Swiss populations. In each population, strains of opposite mating type were found distributed over the whole study site (see Additional file 2). Strains of different mating types were regularly isolated from the same grid points indicating that they can be found in close physical proximity (Table 3). In addition, measures of association [Q] between opposite mating types for significant spatial structures ranged from weakly negative to strongly positive (Table 3), meaning that co-occurrence of strains with different mating types is often more frequently observed than expected by chance.

**Table 3:** Spatial distribution of mating types for selected PAC populations

Species	Study site	Number of strains	Fraction of grid points with only one strain	Number of grid points with > 1 strain	Number of grid points and identified mating types			Association	chi-test	significance^1^
					*MAT1-1*	*MAT1-2*	both	[Q]		
*P. europaea*	Bödmeren	28	0.65	6	7	8	2	0.33	18.75	*
	Creux du Van	135	0.26	35	21	15	11	-0.03	4.41	*
	Zürichberg	129	0.24	42	20	21	14	-0.22	3.60	n.s.
	Scatlé	35	0.21	11	2	6	6	0.94	14.54	*
	Derberence	186	0.19	51	9	30	24	-0.01	5.11	*
*P. helvetica*	Zürichberg	40	0.64	10	20	6	2	-0.13	12.16	*
*P. subalpina*	Bödmeren	80	0.46	22	5	25	11	0.49	4.92	*
	Derberence	36	0.70	7	9	12	2	-0.03	12.80	*
	Scatlé	115	0.30	30	17	11	15	0.43	3.61	n.s.
CSP13	Creux du Van	43	0.61	9	7	13	3	0.25	12.06	*

^1 ^* = significant at p < 0.05; n.s. = not significant

### Analysis of selective pressures based on sequence information

Overall values of ω ranged between 0.26 and 0.46 for the three *MAT *genes and proportions of amino acid sites under purifying selection (ω < 1) were larger than proportions of sites reported under neutral selection (ω = 1) or positive, diversifying selection (ω > 1). For all three *MAT *genes, models that allow codons to evolve under positive selection (M2a and M8) did not fit the data significantly better than models that do not permit positive selection (M1a and M7) (Table 4). Although for the genes *MAT1-1-3 *and *MAT1-2-1 *the log likelihood values of Model M2a and M8 were higher than the associated models M1a and M7, the *p*-values of the LRT statistic were > 0.05. Nevertheless, for *MAT1-1-3 *and *MAT1-2-1 *the LRT test of M0 versus M3 was significant, suggesting variable selection pressure among sites. For these two genes a proportion of sites with ω > 1 was found in models M2a and M8, i.e. 7.6% of the sites with ω = 2.72 for *MAT1-1-3 *and 3.1% of the sites with ω = 5.76 for *MAT1-2-1*. Up to seven amino acid sites were identified as sites of positive selection using the BEB analysis in *MAT1-1-3 *but only one site had strong support with BEB posterior probability > 95% in model M8 (see Additional file 3). In *MAT1-2-1 *five amino acid sites were reported under positive selection but had weak support with BEB posterior probabilities. In *MAT1-1-1*, no proportions of sites with ω > 1 were reported.

**Table 4:** Likelihood ratio tests comparing models of molecular evolution of *MAT *genes

Gene	Analysis	λ^1^	df^1^	2Δλ^1^	*p*-value	d_N_/d_S_	Parameters
*MAT1-1-1*	M0: one ratio	-2205.4733	4	5.9586	0.2023	0.4280	ω = 0.4280
	M3: discrete (κ = 3)	-2202.4940				0.4462	p_0 _= 0.3732, ω_0 _= 0.7052; p_1 _= 0.3932, ω_1 _= 0.1117; p_2 _= 0.2336, ω_2 _= 0.7052
	M1a: nearly neutral	-2202.6642	2	0.0000	1.0000	0.4599	p_0 _= 0.6556, ω_0 _= 0.1761; p_1 _= 0.3440, ω_1 _= 1
	M2a: positive selection	-2202.6642				0.4599	p_0 _= 0.6556, ω_0 _= 0.1761; p_1 _= 0.2332, ω_1 _= 1; p_2 _= 0.1109, ω_2 _= 1
	M7: β	-2202.5615	2	0.0000	1.0000	0.4507	p_0 _= 0.4117; q = 0.5019
	M8: β & ω > 1	-2202.5615				0.4507	p_0 _= 0.1000 p = 0.4117 q = 0.5019; (p_1 _= 0.00001) ω = 1.0000
*MAT1-1-3*	M0: one ratio	-1706.3379	4	20.5175	0.0004*	0.295	ω = 0.2951
	M3: discrete (κ = 3)	-1696.0792				0.3103	p_0 _= 0.0007, ω_0 _= 0.1116; p_1 _= 0.9232, ω_1 _= 0.1117; p_2 _= 0.0761, ω_2 _= 2.7212
	M1a: nearly neutral	-1697.3663	2	2.5742	0.2761	0.2598	p_0 _= 0.7402, ω_0 _= 0; p_1 _= 0.2598, ω_1 _= 1
	M2a: positive selection	-1696.0792				0.3103	p_0 _= 0.9239, ω_0 _= 0.1117; p_1 _= 0, ω_1 _= 1; p_2 _= 0.0761, ω_2 _= 2.7212
	M7: β	-1697.6108	2	3.0594	0.2166	0.3000	p = 0.0050; q = 0.0119
	M8: β & ω > 1	-1696.0811				0.3104	p_0 _= 0.9244 p = 12.5791 q = 99.0000; (p_1 _= 0.0756) ω = 2.7290
*MAT1-2-1*	M0: one ratio	-2133.8605	4	11.1677	0.0247*	0.3714	ω = 0.37135
	M3: discrete (κ = 3)	-2128.2767				0.4212	p_0 _= 0.3531, ω_0 _= 0.2495; p_1 _= 0.6157, ω_1 _= 0.2496; p_2 _= 0.0312, ω_2 _= 5.7427
	M1a: nearly neutral	-2129.6740	2	2.7948	0.2472	0.3528	p_0 _= 0.6472, ω_0 _= 0; p_1 _= 0.3528, ω_1 _= 1
	M2a: positive selection	-2128.2767				0.4212	p_0 _= 0.9688, ω_0 _= 0.2496; p_1 _= 0, ω_1 _= 1; p_2 _= 0.0312, ω_2 _= 5.7428
	M7: β	-2129.8169	2	3.0800	0.2144	0.3387	p = 0.0170; q = 0.0321
	M8: β & ω > 1	-2128.2769				0.4212	p_0 _= 0.9690 p = 33.0717 q = 99.0000; (p_1 _= 0.0310) ω = 5.7612

Models of neutral evolution (M1a and M7) are tested against models that incorporate positive selection (M2a and M8). Comparison of M0 vs. M3 tests variable dN/dS rate ratios among sites. Model M3 classifies sites in the sequence into discrete classes (κ = 3) with both the dN/dS rate ratio ω_0_, ω_1_, ω_3 _and the proportions p_0_, p_2 _and p_3 _estimated from the data. For models M1a and M2a, values of p_0_, p_1_, and p_2_, are the proportions of sites inferred to be evolving under purifying selection, neutral evolution, and positive selection, respectively, and ω_0_, ω_1 _and ω_2 _are their corresponding dN/dS rate ratios. For models M7 and M8, the β distribution, β (p, q), describes the distribution of the dN/dS rate ratio between zero and one, and p_0 _is the proportion of sites within this distribution. In these models, p_1 _is the proportion of sites inferred to be under positive selection, and ω is the dN/dS rate ratio for those sites.

## Discussion

Members of the *Phialocephala fortinii *s.l. - *Acephala applanata *species complex (PAC) have no known sexual state. In this study, however, footprints of sexual reproduction were found in populations from large global samples, providing evidence that sexual reproduction may occur in these fungal species.

### *MAT *locus structure and evolution of homo- and heterothallism in the PAC species

Homothallic fungi can fertilize themselves whereas heterothallic fungi depend on another compatible individual for sexual reproduction to occur. In filamentous ascomycetes the *MAT *locus is the key determinant of breeding system and it has been shown that conversions between heterothallism and homothallism can be achieved by manipulating the *MAT *locus [41,49]. For example, the heterothallic *Neurospora crassa *was capable of self-fertilization after a strain was made carrying both mating types [50,51]. In contrast, a strain of the homothallic *Giberella zeae *was converted to self-sterility after those *MAT *genes were deleted that are present on opposite mating types in other closely related heterothallic species [49,52].

The structure of the *MAT *locus was mapped for 11 additional PAC species for which the *MAT *locus had not yet been characterized. All of these species possessed either the *MAT1-1 *or *MAT1-2 *idiomorph consistent with a heterothallic organization structure. The identified *MAT *genes *MAT1-1-1 *and *MAT1-3-1 *or *MAT1-2-1 *in the respective idiomorphs were of consistent lengths and their arrangement within the *MAT *locus and orientation with respect to each other was the same as found for other PAC species [44] and the other closely related helotialean species *Pyrenopeziza brassicae, Oculimacula yallundae *and *Rhynchosporium secalis *[45,46,53]. The only PAC species containing a homothallic *MAT *locus structure remains *A. applanata *[44]. A controversial topic in fungal biology is whether heterothallism or homothallism represents the ancestral state [37,41,49,54,55]. The question has been addressed in particular for the ascomycete genera *Cochliobolus *[40], and references therein] and *Aspergillus *[56], and references therein] that comprise heterothallic and homothallic species. In the heterothallic *Cochliobolus *species the *MAT *locus structure is conserved as strains are either *MAT1-1 *or *MAT1-2 *and contain the genes *MAT1-1-1 *or *MAT1-2-1 *with the same gene orientation within idiomorphs. In contrast, in the homothallic species the *MAT *locus structure is unique because single individuals possess both *MAT *genes that are either fused into a single ORF, are closely linked or likely present on different chromosomes. Due to this variation in *MAT *locus structure and phylogenetic evidence of independent evolution of self-fertility, the likely derived state in this genus was proposed to be homothallism [49]. Recombination is normally suppressed in the *MAT *locus because the sequences of the two idiomorphs are significantly diverged [57,58]. However, small identity island of 8 and 9 nucleotides were identified within *MAT *genes of the heterothallic *Cochliobolus heterostrophus *and rare recombination events between these small identity islands were suggested to be the likely mechanism for the conversion of reproductive modes in ancestral lineages [49]. In contrast, the prevalence of homothallic species, phylogenetic analyses and comparisons of genome sequences suggest that heterothallism is the derived state in the genus *Aspergillus *[59] albeit additional characterizations of *MAT *loci of supposedly asexual *Aspergillus *species may alter this view [56].

For PAC species both evolutionary trajectories of reproductive modes from homothallism to heterothallism and *vice versa *are conceivable. Because *MAT *genes control reproductive processes the information gained by comparing their DNA sequences might allow reconstructing the evolutionary history of a species complex and indicate mechanisms that led to changes in reproductive modes [49]. The basal position of *A. applanata *in the *MAT *gene phylogenies suggests that the *MAT *genes of *A. applanata *are older than the *MAT *genes of the remaining PAC species which are grouped in clusters, and thus are more recently derived and share a common ancestor (see Additional file 4). In fact, this finding is congruent with population genetic data and other phylogenetic data based on DNA sequences of housekeeping genes and RFLP loci that showed that *A. applanata *is the most diverging species in the PAC [60]. It is possible that a switch in reproductive mode of a homothallic ancestor was the source of the strong divergence between *A. applanata *and other PAC species and that thus, *A. applanata *represents an ancient lineage within the PAC. In populations of an ancestral homothallic species a structural change in the *MAT *locus, e.g. after a deletion of a *MAT *gene could have provoked a switch to heterothallism and created barriers to self-mating. These genetic barriers could have increased and maintained genetic diversity, eventually leading to speciation and radiation that gave rise to the current heterothallic species. The likely mechanisms that have initiated such a switch in the PAC might be explained by re-arrangements among idiomorphs and the influence of transposable elements on recombination events in the *MAT *locus of *A. applanata *[44]. On the other hand the heterothallic PAC species possess a conserved *MAT *locus structure with congruent *MAT *gene orientation and position within the *MAT *locus. This organization is also consistent with the *MAT *locus structure found in closely related helotialean species that are heterothallic [45,46,53]. Thus, the prevalence of structurally heterothallic species in the PAC and their conserved *MAT *locus organization are arguments in favor of heterothallism as ancestral breeding system.

### Mating type distribution and gametic equilibrium

If sexual reproduction plays an important role in the life cycle of a fungal species, natural populations would be expected to show following characteristics [16,19]: (i) 1:1 ratio of mating types because frequency-dependent selection acts on the rare mating type that has the higher chance for mating, and (ii) gametic equilibrium at unlinked loci because under random mating sexual recombination will reduce non-random association of alleles. Both criteria were met for the majority of PAC populations. The 6 out of 52 populations that displayed gametic disequilibrium were restricted to the species *P. subalpina*. Independently of the reproductive mode several factors can cause non-random association of alleles that result in gametic disequilibrium. These factors include high frequency of rare alleles, genetic drift, population admixture and selection [16,61]. We therefore re-analyzed these collections of *P. subalpina *after pooling rare alleles but the results did not change (data not shown). We then tested whether traces of population admixture could be identified in the two populations using the program STRUCTURE [62] and evidence for admixture was found in two populations. Moreover, the strains collected from Bialowieza could be allocated with high posterior probabilities to two subpopulations and the index of association I_A _did not significantly deviate from zero in both of these subpopulations (data not shown).

The spatial distribution of mating type within study sites is often neglected in studies aiming at assessing the reproductive biology of fungal species. In this study opposite mating types were often collected at the same grid points within study sites. Moreover, the measures of spatial association Q were predominantly neutral to strongly positive, meaning that for several populations the occurrence of both mating types at the same grid point was higher than expected by chance alone. Thus, the physical proximity of compatible mating types in the study sites meets another prerequisite for successful sexual reproduction in the PAC.

### Selection acting on mating type genes in PAC

If *MAT *genes play an important role in the initiation and further steps of sexual reproduction [39,40] they are expected to be functionally conserved in sexual species. All PAC species possessed structurally identical idiomorphs and translation of the *MAT *genes suggested that they encode functional proteins. In *A. applanata *the insertion of a transposable element in the 3' end of *MAT1-2-1 *caused a truncation of 30 amino acids in the translated protein [44]. Moreover, insertions of 3 and 9 bp were found in *MAT1-1-1 *of *A. applanata *and in *MAT1-2 *deletions as long as 18 bp were found for cryptic species CSP12, CSP14, *P. letzii, P. europaea *and *A. applanata*. However these indels did not result in stop codons or frame shifts that could affect functionality.

The molecular analyses revealed that all three *MAT *genes appear to be under strong purifying selection because sites under purifying selection were predominant. The overall values of ω obtained ranged between 0.26-0.46, and the proportions of sites under purifying selection (ω < 1) were much larger than the fraction evolving neutrally or under positive selection accounting for at least 65% of the sites. In only few studies the selective pressures on *MAT *genes were analyzed and similar overall values of ω were found ranging between 0.14 and 0.49 in sexual or presumed sexual fungi [22,23,25,26]. The predicted effect of long-term asexuality is the decay of genes specific for sex and recombination due to the relaxation of selective constraints [63] but the results of our study suggest that *MAT *genes in PAC species are highly conserved and functional.

Rapid divergence of reproductive proteins, due to positive selection and adaptive evolution, is believed to be important in the formation of reproductive barriers leading to speciation [64-66]. Competition for gamete recognition between individuals might explain the rapid evolution of reproductive proteins [67]. High levels of interspecific polymorphism have been found in *MAT *genes [22,68]. In our study the amount of polymorphism between the structurally heterothallic PAC species was also high and ranged between 8.7 and 9.4%. This was in the range found for non-coding RFLP loci and approximately 2.5-fold more than in levels found in the housekeeping gene beta-tubulin [60]. However, statistical evidence of positive selection of *MAT *gene, i.e. high levels of non-synonymous substitutions leading to changes in *MAT *proteins, has only been shown in the genus *Neurospora *[25]. Our results showed that models of evolution that allow positive selection (ω > 1) were not more likely than those incorporating neutral evolution. Nevertheless, the comparison of model M0 vs. M3 tested significant for variable pressure among sites in *MAT1-1-3 *and *MAT1-2-1 *and up to seven sites evolving under positive selection were identified in these genes, although only one was significant in the BEB analysis. It might be assumed that these and many more sites evolve under positive selection in *MAT *genes but that, in a strong background of purifying selection, they remain uncovered by the currently available analysis methods. However, whether the putative protein alterations at such positively selected sites have a biological significance is unknown.

### Have PAC species a sexual life cycle?

In the present study we combined different indirect approaches to analyze the importance of sexual reproduction in the PAC. The population analyses were in accordance with random mating and sexual recombination in abundant collections from different ecosystems. Moreover, the presence of a conserved *MAT *locus in all PAC species suggests that *MAT *genes are functional and are still involved in mating processes. Yet, although the data does not allow rejecting the hypothesis of sexual recombination, the confirmation of the teleomorph *in vitro *is pending. Our first attempts to induce apothecia formation of PAC species failed when we tried to cross opposite mating types under laboratory conditions [44]. However, *in vitro *induction of the teleomorph is often difficult and time consuming and some perseverance is required [17,18]. Nevertheless, we successfully induced teleomorph formation of *Phaeomollisia piceae*, the closest known relative of the PAC [69]. *P. piceae *forms small darkly pigmented apothecia of less than 800 μm diameter. Considering that PAC species might form similar apothecia in the soil or on roots it seems likely that the teleomorph has been overlooked in field studies.

## Conclusions

In this study we evaluated the importance of sexual reproduction in the PAC, the dominant dark septate root endophytes of woody plant species, and showed that the signature of sex is present in all of these putatively asexual species. The *MAT *locus resembles those of closely related heterothallic ascomycetes, is conserved and purifying selection likely preserves functionality of its genes. The hypothesis of sex occurring in the PAC cannot be rejected based on the random association of alleles at multiple loci, the equal mating type frequencies and the spatial distribution of opposite mating types in populations from different ecological habitats, hosts and continents. We believe that further field studies and *in vitro *crosses will finally lead to the discovery of the sexual state, which is the missing link to better understand how reproductive biology shaped the evolutionary history and influences the ecological role of the PAC.

## Methods

### Collection of PAC species and mating type screening

A total of 3, 639 strains derived from 33 study sites were included in the mating type screening (Table 2). These strains represent 19 PAC [32] species eight of which are formally described [28,29]. Several classes of molecular markers were developed for PAC species assignment including PCR fingerprints, single-copy restriction fragment length polymorphisms (RFLP), multi-locus DNA sequences, and microsatellites. Each of these molecular markers supported the delineation of multiple species in this complex, with concordant cryptic species defined by all markers [60,70]. PAC species communities were either collected on grid nets (196 m^2^, 74 grid points) [33] or on transects (50 m, 11 grid points) [32] from Europe (number of study sites n = 19), North America (n = 10) and Asia (n = 4). Collections included both undisturbed and managed forests.

### Multiplex PCR

A multiplex PCR method was developed to discriminate between isolates carrying either the *MAT1-1 *or the *MAT1-2 *idiomorph based on available sequence data of the *MAT *locus of seven structurally heterothallic PAC species [44]. The primer Pf_HMG_R.03 (5'-TCCTCGAACCGGTGCCACGAATGACTCCGA-3') was designed to match a flank of the idiomorphic region that is conserved between the two mating types of these species. The *MAT1-1 *specific primer (Pf_HMG_F4: 5'-AGCTGAGCTCGAGACCTCGTTCGC-3') and a *MAT1-2 *specific primer (Pf_MAT1-1F1c: 5'-CTTGTACGGTCCGGCAATCCACA-3') were designed to generate DNA fragments of distinct length in combination with Pf_HMG_R.03 that could be easily differentiated on agarose gels. All PCRs were performed on a Biometra T1 thermal cycler in a 10 μl reaction volume containing approximately 2 ng template DNA, 5 pmol of each primer, 50 mM KCl, 10 mM Tris-HCl, 1, 5 mM MgCl2, 200 μM dNTPs (Promega AG, Dübendorf) and 0.2 U Taq polymerase (Promega AG, Dübendorf). Cycling conditions during PCR were 2 min at 94°C followed by 29 cycles of denaturation for 20 s at 94°C, annealing for 30 s at 60°C and extension for 60 s at 72°C, followed by a final extension step of 5 min at 72°C.

### Mating type frequencies in PAC populations

The number of strains from a single study site and belonging to the same PAC species is here defined as a population. Before assessing significance of deviation from a 1:1 mating type ratio in a population, each population was clone-corrected using 12 microsatellite loci [70] or 11 single-copy RFLP markers [33], meaning that only one representative of each multi-locus genotype was included. Clone-correction is necessary because the same fungal thallus is found on several grid points. Five species [cryptic species (CSP) 8, 9, 10, 18 and 19] occurred at exceptionally low frequencies in the collection sites and represented less than 3% of all collected strains. For these species, the distribution of mating types was assessed after pooling all strains of a species, irrespective of the study site from which they were isolated. Significance was tested using the exact binomial test. In addition, Bonferroni corrections were performed for multiple comparisons.

Because species abundance distributions within local PAC communities follow a hyperbolic distribution with a few very abundant species and many "rare" species [48], clone-corrected datasets for such rare species resulted in a low number of distinct genotypes (< 10). In these cases, only the frequency of mating types was recorded without testing for significance.

### Analysis of gametic disequilibrium

Significant deviations from multi-locus gametic equilibrium in a population were tested using the index of association I_A _and its variance according to Maynard Smith et al. [47]. All calculations of I_A _were based on clone-corrected datasets and multi-locus genotypes with missing data were excluded from the analysis.

### Spatial distribution of mating types in selected study sites

Spatial distribution of mating types was analyzed for five Swiss study sites. Only the two most abundant PAC species per community were included in this analysis. For each of these species, the number of grid points was recorded where either *MAT1-1 *or *MAT1-2 *or both mating types were found. A contingency table test for independence of the spatial distribution of the mating types was performed and a measure of association between *MAT1-1 *and *MAT1-2 *(*Q*) was calculated as described in Pielou [71].

### Studying the presence of both mating types in single isolates

In few cases, no amplification product was obtained in some strains, or both the *MAT1-1 *and *MAT1-2 *specific fragments were amplified in single strains. In order to study the reason for these observations, ten new single-hyphal tip cultures were prepared from the original slants of the three strains having displayed both PCR products and of the five strains from which no PCR product was obtained. After harvesting and extracting DNA of mycelia from both the original slants and the newly prepared single-hyphal tip cultures, the mating types were re-determined using the multiplex PCR.

### Sequencing the *MAT locus*

The *MAT *locus was sequenced for 11 PAC species and compared to the *MAT *locus structure of 8 previously sequenced PAC species [44]. For each of these species the *MAT *locus of both the idiomorph *MAT1-1 *and *MAT1-2 *was sequenced (Table 1). The detailed sequencing strategy and the sequencing primers used are given in the Additional files 5 &6, and the *MAT *locus was annotated as described in [44]. PCR fragments were directly purified using an "ExoSap" protocol [70]. Cycle sequencing was performed with the Big-Dye v3.1 kit (Applied Biosystems) in a 10 μl reaction volume using 1 μl purified PCR amplification product, 0.5 μl BigDye v3.1, 1.9 μl 5 × Buffer, 5.6 μl ddH2O and 1 μl of the sequencing primer (10 μM). Cycle sequencing reactions were performed on a Biometra T1 thermal cycler with the following running conditions: 60 s at 96°C followed by 55 cycles of 10 s at 95°C, 5 s at 50°C and 4 min at 60°C. Dye-labeled fragments were cleaned using BigDye Xterminator Purification kit following manufacturer's instructions (Applied Biosystems). Samples were run on an ABI 3130xl DNA Analyser (Applied Biosystems).

### Analysis of selective pressures

In order to assess what kind of selective pressure is acting on any of the three *MAT *genes a maximum likelihood (ML) method implemented in the program CODEML in the PAML 4.1 software package [72] was used to compare the rate of non-synonymous substitutions with the rate of synonymous substitutions (d_N_/d_S _= ω). Under neutrality ω is expected to be 1. When amino acid changes are favored, indicative of positive selection, ω is expected to be > 1, whereas under purifying selection amino acid changes are prevented and ω is expected to be < 1. Positive selection of small number of codons in the *MAT *genes may be masked when the entire gene is strongly affected by purifying selection. Therefore, a codon by codon approach was used based on the ML method implemented in CODEML that allows site by site identification of particular codons that have been evolving under repeated and strong positive selection [72]. The three *MAT *genes were analyzed separately and a gene tree describing the phylogenetic relationships of all taxa studied (one strain per species) was constructed using the ML method implemented in the program treefinder [73] using the best-fitting mutation model. The inferred trees served as the basis for the implementation of the ML methods in the CODML (see Additional file 4).

Three sets of models, commonly applied to test hypotheses of selection, were used. Following the suggestions of Yang [72] the site model pairs that appear to be particularly useful for real data analysis, are the M1a (nearly neutral) versus M2a (selection) and M7 (neutral) versus M8 (selection). However, model M0 (one-ratio) was also compared versus M3 (discrete) in order to see if the selective pressure is variable among sites. Because the comparison of M7 versus M8 is less conservative, this set of models may indicate positive selection even when none is detected by the M1a vs. M2a comparison.

The strength of positive selection was calculated using the Likelihood Ratio Test (LRT) [72] implemented in PAML by comparing twice the log likelihood difference in a chi-square test. The degree of freedom for the test statistic is determined by the difference in estimated parameters between the models of selective pressures being compared, i.e. four [M0 (one-ratio) vs. M3 (discrete)] or two [M1a (nearly neutral) vs. M2a (selection), and M8 (selection) vs. M7 (neutral)] degrees of freedom [72]. Codons that are identified as having evolved under positive selection have high posterior probabilities (*p *> 0.95). Posterior probabilities for such sites were estimated based on Bayes Empirical Bayes (BEB) analysis [74].

The *MAT *gene sequence of the homothallic *A. applanata *species was not included in this analysis because it would have inflated the alignments with deletions. For example, the *MAT1-2-1 *sequence of *A. applanata *contained a large deletion of 90 bp at its 3' end, due to the insertion of a transposable element in the *MAT *locus of the species [44]. Gaps in alignments cannot be handled by CODEML and the program automatically removes such sites. Thus, in order to prevent the loss of informative sites in the sequence of the other species, the *A. applanata *sequences were removed from the alignment prior to the analyses.

## List of abbreviations

*MAT*: mating type; PAC: *Phialocephala fortinii *s.l. - *Acephala applanata *species complex.

## Authors' contributions

PZ participated in the design of the study, conducted the analysis of selective pressures, drafted and wrote the manuscript. VQ participated in the molecular work and conducted microsatellite analysis. AD carried out molecular work and participated in the sequence alignment. CG designed and coordinated the study, performed statistical analysis and helped to draft the manuscript. All authors read and approved the final manuscript.

## Supplementary Material

Additional file 1 **Testing the presence of both idiomorphs in selected PAC strains**. Testing the presence of both idiomorphs, or the absence of *MAT *genes in selected PAC strains. Click here for file

Additional file 2 **Spatial distribution of mating types**. Example of the spatial distribution of mating types for a population of *Phialocephala subalpina *and *Phialocephala turicensis*. Click here for file

Additional file 3 **Sites in MAT genes identified to evolve under positive selection**. Sites under positive selection were identified using the Bayes Empirical Bayes analysis under different CODML site models in the PAML software package. Click here for file

Additional file 4 **Phylogenies inferred from *MAT *genes**. Phylogenies inferred for the three genes *MAT1-1-1, MAT1-1-3*, and *MAT1-2-1*. The sequence of *Acephala applanata *served as outgroup in all three analysis. Click here for file

Additional file 5 **Sequencing strategy for *MAT *idiomorphs**. Sequencing strategy applied to sequence the complete *MAT *idiomorphs for PAC species. Sequences of primers are given in the Additional file 5. Click here for file

Additional file 6 **Sequencing primers**. Sequences of primers used to sequence the complete *MAT *idiomoprh of PAC species. Click here for file
